# Placental Inflammasome mRNA Levels Differ by Mode of Delivery and Fetal Sex 

**DOI:** 10.3389/fimmu.2022.807750

**Published:** 2022-03-25

**Authors:** Anya L. Arthurs, Melanie D. Smith, Mhyles D. Hintural, James Breen, Dylan McCullough, Francesca I. Thornton, Shalem Y. Leemaqz, Gustaaf A. Dekker, Tanja Jankovic-Karasoulos, Claire T. Roberts

**Affiliations:** ^1^ Pregnancy Health and Beyond Laboratory, Flinders Health and Medical Research Institute, Flinders University, Adelaide, SA, Australia; ^2^ South Australian Genomics Centre, South Australian Health & Medical Research Institute, Adelaide, SA, Australia; ^3^ Adelaide Medical School, University of Adelaide, Adelaide, SA, Australia

**Keywords:** placenta, inflammation, pregnancy, labour, parturition, inflammasome

## Abstract

Parturition signals the end of immune tolerance in pregnancy. Term labour is usually a sterile inflammatory process triggered by damage associated molecular patterns (DAMPs) as a consequence of functional progesterone withdrawal. Activation of DAMPs recruits leukocytes and inflammatory cytokine responses in the myometrium, decidua, cervix and fetal membranes. Emerging evidence shows components of the inflammasome are detectable in both maternal decidua and placenta. However, the activation of the placental inflammasome with respect to mode of delivery has not been profiled. Placental chorionic villus samples from women delivering at term *via* unassisted vaginal (UV) birth, labouring lower segment caesarean section (LLSCS, emergency caesarean section) and prelabour lower segment caesarean section (PLSCS, elective caesarean section) underwent high throughput RNA sequencing (NextSeq Illumina) and bioinformatic analyses to identify differentially expressed inflammatory (DE) genes. DE genes (*IL1RL1*, *STAT1*, *STAT2*, *IL2RB*, *IL17RE*, *IL18BP*, *TNFAIP2*, *TNFSF10* and *TNFRSF8*), as well as common inflammasome genes (*IL1B*, *IL1R1*, *IL1R2*, *IL6*, *IL18*, *IL18R1*, *IL18R1*, *IL10*, and *IL33*), were targets for further qPCR analyses and Western blotting to quantify protein expression. There was no specific sensor molecule-activated inflammasome which dominated expression when stratified by mode of delivery, implying that multiple inflammasomes may function synergistically during parturition. Whilst placentae from women who had UV births overall expressed pro-inflammatory mediators, placentae from LLSCS births demonstrated a much greater pro-inflammatory response, with additional interplay of pro- and anti-inflammatory mediators. As expected, inflammasome activation was very low in placentae from women who had PLSCS births. Sex-specific differences were also detected. Placentae from male-bearing pregnancies displayed higher inflammasome activation in LLSCS compared with PLSCS, and placentae from female-bearing pregnancies displayed higher inflammasome activation in LLSCS compared with UV. In conclusion, placental inflammasome activation differs with respect to mode of delivery and neonatal sex. Its assessment may identify babies who have been exposed to aberrant inflammation at birth that may compromise their development and long-term health and wellbeing.

## Introduction

Pregnancy is a time of immunological challenge and change. Implantation and placentation are defined as pro-inflammatory phases, followed by an anti-inflammatory stage associated with fetal development and growth. Finally, parturition signals the end of immunological tolerance in pregnancy, indicating another pro-inflammatory stage which mediates labour (1). Although inflammation at the fetomaternal interface is often associated with pregnancy complications (2), inflammation and the expression of pro-inflammatory cytokines in the uterus are critical for cervical ripening and labour initiation for healthy delivery (3–6).

Labour at term follows functional progesterone withdrawal, inducing a normally sterile inflammatory process characterised by signals of cellular stress (damage-associated molecular patterns [DAMPs]). DAMPs, often expressed in response to rupture of membranes, are recognised by pattern recognition receptors (PRRs), leading to innate immune system activation of the inflammasome (7–9). The inflammasome is a cytosolic multi-subunit protein complex that consists of a sensor molecule, typically a PRR, an adaptor protein apoptosis-associated speck-like protein containing a caspase recruitment domain (ASC), and pro-CASP-1 (7–9). While there are multiple inflammasome pathways, five have been well-characterised; NLR and pyrin domain-containing protein (NLRP)-1 (10); NLRP3 (11); NLR family caspase activation and recruitment domain (CARD) domain-containing protein-4 (NLRC4) (12, 13); absent in melanoma-2 (AIM2) (14); and pyrin (15).

Activation of the nuclear factor kappa B (NF-κB) pathway leads to assembly of the inflammasome complex, as well as independently leading to increased pro-interleukin (IL)-1β and pro-IL-18 expression (9, 16, 17). Assembly of the inflammasome complex leads to a proteolytic inflammatory cascade mediated by cleavage of procaspase-1 (pro-CASP-1) into its active form, CASP-1, which cleaves pro-IL-1β and pro-IL-18 into the active cytokines, IL-1β and IL-18 (7, 8, 16, 18), contributing to the labour cascade. For this reason, IL-1β and IL-18 can be measured as markers of inflammasome activation.

Key molecules in the inflammasome, plus generalised inflammatory pathways, have been localised to the chorioamniotic membranes, in which spontaneous term labour is associated with greater inflammasome assembly than those without spontaneous labour (7). Significantly increased concentrations of CASP-1 and IL-1β were also identified in amniotic fluid of women who underwent spontaneous labour at term compared to women who did not undergo labour at term (19). Additionally, it has been suggested that inflammasome overactivation can also contribute to the progression of pregnancy complications such as preeclampsia (8, 20–24), premature rupture of membranes (PROM) (25), fetal growth restriction and preterm labour (9).

Whilst characterising the expression of inflammasome-related molecules has mostly been confined to the chorioamniotic membranes and the amniotic fluid, not the placenta, there is evidence to suggest that the placenta can serve as a proxy for uterine inflammation (5, 26) (see *Discussion*).

Even for term births, delivery can be spontaneous and unassisted, operative vaginal delivery, in labour emergency caesarean section or planned prelabour caesarean section. The latter can be for medical indications but there are increasing numbers planned for non-medical reasons. It is not known whether these different modes of delivery affect the placental inflammasome. In this study, we profiled the expression of these pro-inflammatory cytokines, as well as other inflammatory molecules, in the placentae of women who delivered *via* unassisted vaginal birth (UV), emergency caesarean section [labouring lower segment caesarean section (LLSCS)] and elective caesarean section [prelabour lower segment caesarean section (PLSCS)]. Elucidating the inflammasome activation in the placentae from women with different modes of delivery would allow inferences to be made regarding potential overactivation of the inflammasome and an exaggerated inflammatory response in certain modes of delivery and, in turn, the potential effects on mothers and their neonates.

## Materials and Methods

### Tissue Samples

Term placentae were obtained at the Lyell McEwin Hospital in Elizabeth, South Australia, from women recruited as part of the SCreening fOr Pregnancy Endpoints (SCOPE) (2005–2008) and Screening Tests to predict poor Outcomes of Pregnancy (STOP) (2015–2018) cohort studies (27). Both SCOPE and STOP studies were registered with the Australian and New Zealand Clinical Trials Registry (ACTRN 12607000551493 and ACTRN 12614000985684, respectively).

All placentae were associated with uncomplicated pregnancies and defined by the following methods of delivery: UV, LLSCS, and PLSCS. Samples from LLSCS deliveries underwent emergency Caesarean section due to failure to progress in labour, and/or detection of fetal distress (lowered heartrate or presence of meconium). Tissue biopsies from placentae were washed in phosphate-buffered saline (PBS) before being submerged in RNAlater Stabilisation Solution (Thermo Fisher Scientific, Massachusetts, USA) and stored at -80°C. Ethics approvals were obtained from the Queen Elizabeth Hospital Human Research Ethics Committee (TQEH/LMH HREC/1712/5/2008; SCOPE) and Women’s and Children’s Health Network Human Research Ethics Committee (HREC/14/WCHN/90; STOP). All women provided written informed consent.

### RNA Extraction

Term placental tissues (0.25g) were weighed and washed with phosphate-buffered saline (PBS). Tissue was disrupted by homogenizing for 3.5 mins at 30 Hz (TissueLyser, QIAGEN) in 600 μL Buffer RLT Plus (RNeasy Plus Mini Kit; QIAGEN, Victoria, Australia). Total RNA was extracted from the supernatant using the RNeasy Plus Mini Kit (QIAGEN) according to the manufacturer’s protocol. The purity and integrity of extracted RNA samples were determined using the Experion™ (BioRad, New South Wales, Australia) and samples used had a RIN ≥ 8.

### Sequencing and Bioinformatic Analyses

High Throughput Sequencing was performed at Flinders University using a NextSeq Illumina sequencer. Alignment was performed using BWA version 0.7.17-r1188 (GRCh37) and output generated in FASTQ format (28). Quality control metrics were assessed using FastQC (http://www.bioinformatics.babraham.ac.uk/projects/fastqc/) to check for per base sequence quality, sequence length distribution and duplication levels. Differential expression analyses were conducted in the R statistical environment (v.4.0.5), using the *edgeR* (v.3.16.5) (29) and *limma* (v.3.30.11) (30) packages. Briefly, *edgeR* was used to filter mRNA with low expression and normalise for library composition bias. All samples were then normalised using the Trimmed Mean of M values (TMM). Sample-weights and log transformation were performed using the *limma* package with the voom function used to estimate the mean-variance relationship between individual observations and then applied to the normalised log-counts data. Differential expression analyses including moderated F-statistic evaluation, adjusted p-value estimation and log_2_ fold change analysis were performed using a moderated t-test (31) with Benjamini-Hochberg (BH) multiple hypothesis test corrections (32). After adjustment, gene expression was considered significantly different at FDR ≤ 0.05.

### cDNA Synthesis and Quantitative Polymerase Chain Reaction (qPCR)

Synthesis of complementary DNA (cDNA) was conducted beginning with 1μg of total RNA using the QuantiNova Reverse Transcription Kit (QIAGEN) according to the manufacturer’s protocol. qPCR was conducted with SYBR Green (QIAGEN) according to manufacturer’s instructions, with YWHAZ and β-actin as housekeeping genes (primer sequences in **Table 1**). Denaturation was performed at 95°C for 10 secs, annealing at 53°C for 45 secs, and extension at 72°C for 30 secs, for a total of 50 cycles. qPCR results were analysed using the 2^-ΔΔCT^ method (33). Samples were sorted into groups depending on mode of delivery (see **Table 2** for numbers and characteristics of women’s and infants’ samples used).

**Table 1:** Sequences for qPCR primers.

Gene	Forward primer	Reverse primer
*IL1B*	CCACAGACCTTCCAGGAGAATG	GTGCAGTTCAGTGATCGTACAGG
*IL1R1*	GTGCTTTGGTACAGGGATTCCTG	CACAGTCAGAGGTAGACCCTTC
*IL1R2*	GGCTATTACCGCTGTGTCCTGA	GAGAAGCTGATATGGTCTTGAGG
*IL18*	GATAGCCAGCCTAGAGGTATGG	CCTTGATGTTATCAGGAGGATTCA
*IL18BP*	GTGTCCAGCATTGGAAGTGACC	GGAGGTGCTCAATGAAGGAACC
*IL18R1*	GGAGGCACAGACACCAAAAGCT	AGGCACACTACTGCCACCAAGA
*IL6*	AGACAGCCACTCACCTCTTCAG	TTCTGCCAGTGCCTCTTTGCTG
*IL2RB*	GGTGGAACCAAACCTGTGAGCT	GGTGACGATGTCAACTGTGGTC
*IL17RE*	CCTACCTGCAAGAGGACACTGT	CCATCTGAGTGTGCTGGCTGTA
*TNFAIP2*	TGCTCCAGAACCTGCATGAGGA	AACTCAGGCAGCCTCGTGTCTA
*TNFSF10*	TGGCAACTCCGTCAGCTCGTTA	AGCTGCTACTCTCTGAGGACCT
*TNFRSF8*	ATCTGTGCCACATCAGCCACCA	AAGGTGGTGTCCTTCTCAGCCA
*STAT1*	ATGGCAGTCTGGCGGCTGAATT	CCAAACCAGGCTGGCACAATTG
*STAT2*	CAGGTCACAGAGTTGCTACAGC	CGGTGAACTTGCTGCCAGTCTT
*IL10*	TCTCCGAGATGCCTTCAGCAGA	TCAGACAAGGCTTGGCAACCCA
*IL33*	GCCTGTCAACAGCAGTCTACTG	TGTGCTTAGAGAAGCAAGATACTC
*IL1RL1*	CTCTGTTTCCAGTAATCGGAGCC	GCAGCCAAGAACTGAGTGCCTT
*YWHAZ*	ACCGTTACTTGGCTGAGGTTGC	CCCAGTCTGATAGGATGTGTTGG
*ACTINB*	CACCATTGGCAATGAGCGGTTC	AGGTCTTTGCGGATGTCCACGT

**Table 2:** Characteristics of women and infants from the study by mode of delivery (UV, LLSCS, PLSCS) and analytic technique (RNA sequencing, qPCR analysis and Western Blotting).

	RNA sequencing (n = 50 total)				qPCR (n = 70 total)				Western Blotting (n = 24 total)			
Mode of delivery	UV (n = 28)	LLSCS (n = 16)	PLSCS (n = 6)	p-value	UV (n = 40)	LLSCS (n = 20)	PLSCS (n = 10)	p-value	UV (n = 12)	LLSCS (n= 8)	PLSCS (n= 4)	p-value
Maternal age (years)	24.4 ± 3.41	25.6 ± 5.58	27.8 ± 3.37	0.08	25.9 ± 4.15	25.1 ± 4.97	27.2 ± 2.35	0.051	24.5 ± 3.17	25.5 ± 4.98	26.2. ± 1.71	0.08
Maternal BMI (kg/m²)	26.4 ± 5.53	30.2 ± 9.32	29.4 ± 4.04	0.1	24.9 ± 6.02	26.3 ± 7.15	28 ± 5.53	0.054	25.7 ± 4.30	25.9 ± 6.61	30.0 ± 4.91	0.2
Smoking Yes (N %)	23 (82.1)	10 (62.5)	4 (66.7)	0.3	7 (17.5)	4 (20)	0 (0)	0.5	2 (16.6)	1 (12.5)	0 (0)	1.0
Gestational age (weeks)	39.4 ± 1.2	39.2 ± 1.53	37.5 ± 3.02	0.4	39 ± 1.47	39.8 ± 1.61	39.0 ± 2.10	0.2	39 ± 1.99	39.4 ± 1.05	39.0 ± 1.40	0.09
Birthweight (g)	3520 ± 415	3493 ± 470	3435 ± 956	0.8	3601 ± 451	3781 ± 342	3746 ± 592	0.3	3612 ± 216	3607 ± 454	3765 ± 509	0.4
Fetal sex	XX = 20 XY = 8	XX = 9 XY = 7	XX = 3 XY = 3	0.4	XX = 20 XY = 20	XX = 10 XY = 10	XX = 5 XY = 5	1.0	XX = 6 XY = 6	XX = 4 XY = 4	XX = 2 XY = 2	1.0
Fetal distress (N %)	-	-	-	-	5 (12.5)	4 (20)	0 (0)	0.3	-	-	-	-

Modes of delivery: unassisted vaginal (UV), labour lower segment Caesarean section (LLSCS), and prelabour LSCS (PLSCS). Maternal age, maternal BMI, gestational age, and birthweight are presented as mean ± standard deviation. For smoking status (recorded at time of recruitment, 9-16 weeks**’** gestation) the number of smokers is shown, followed by the percentage of smokers in parentheses. For fetal sex, the number of female (XX) and male (XY) infants is shown. All samples used for RNA sequencing were included in qPCR analysis, with the addition of 20 extra samples to validate our findings. A subset of samples used for RNA sequencing were used for Western Blotting, due to quality and quantity of tissue available.

### Western Blotting

#### Protein Extraction

Term placental tissues (0.25g) were weighed into Powerlyzer tubes and disrupted in 400 μL ice cold RIPA (Radio Immuno Precipitation Assay) buffer. Samples (see **Table 2** for numbers and characteristics of women’s and infants’ samples used) were homogenised (3.5 mins at 30 Hz) using the TissueLyser (QIAGEN). After homogenisation, samples were diluted with 400 μL RIPA buffer. Protein concentration was measured *via* a Bradford Assay using the SpectraMax iD5 (Molecular Devices) at 595 nm absorbance, prior to sample storage at -80°C.

#### SDS-PAGE

Samples (25 μg each) were denatured and reduced in Laemmli 4x buffer (GTX16355, GeneTex) and 8% 2-mercaptoethanol for 5 mins at 95°C. Samples were then loaded onto either a 4–20% Mini-PROTEAN^®^ TGX Stain-Free™ Protein Gel (4568094, Bio-Rad) or 4–20% Mini-PROTEAN^®^ TGX™ Precast Protein Gel (4561094, Bio-Rad), along with a molecular weight marker (GTX49384, GeneTex). SDS-PAGE was carried out for 1 h at 100 V in 1x Running buffer (pH 8.3) using a Mini-PROTEAN Tetra Vertical Electrophoresis Cell (Bio-Rad). Stain-Free Protein gels were checked for complete protein separation using the ChemiDoc Touch Imaging System (Bio-Rad).

#### Transfer

Protein was transferred to a PVDF membrane over 16 hours at 27V and 4°C in transfer buffer (25 mM Trizma Base, 190 mM glycine, 20% methanol; pH 8.3) using the Criterion™ blotter (Bio-Rad). Successful transfer of protein was checked by Ponceau S staining of the membrane, as well as either Coomassie Blue staining (for the TGX Precast Protein Gel) or the ChemiDoc Touch Imaging System (Bio-Rad) (for the TGX Stain-Free™ Protein Gel).

#### Blotting

Membrane was blocked in Tris Buffered Saline Tween20 (TBST) with 5% skim milk for 2 h at room temperature. Membrane was then incubated in blocking buffer for 1 h with either IL-1β Polyclonal Antibody (P420B, Invitrogen) at 1:200 dilution, or IL-18 Polyclonal Antibody (PA5-110679, Invitrogen) at 1:300. Membrane was washed 3-4 times with TBST, then incubated with a goat anti-rabbit Immunoglobulins/HRP secondary antibody (P044801-2, Dako) at 1:5000 in the blocking buffer. Membrane was washed 3-4 times in TBST, then incubated in Clarity Western ECL Substrate (1705060, Bio-Rad) for 5 mins and imaged. The density of each band (determined by the ChemiDoc Touch Imaging System) was determined prior to analysis using ImageLab software from Bio-Rad™. Analysis controlled protein loading for each sample by normalizing using stain-free total protein quantification (34) and was further normalized to an internal control sample (pooled term placentae) on each membrane. Samples were run in duplicate and averaged for final analysis.

### Statistical Analysis

Statistical analysis for differences between groups from qPCR and Western Blotting data was undertaken using SPSS Statistics Software. Outliers were removed from the data using a Grubbs’ test. Data were assessed for normality distribution and a two-way ANOVA test was conducted. Differences between groups were considered significant for *p* ≤ 0.05. All tests of statistical significance are two-sided, and adjustments were made for multiple comparisons.

## Results

### Cohort Characteristics With Respect to Mode of Delivery (UV, LLSCS or PLSCS)

RNA sequencing data was available for 50 placentae from uncomplicated pregnancies where mode of delivery was known. Mode of delivery and birth characteristics are presented in **Table 2**.

### Genes Are Differentially Expressed in a Sex-Specific Manner in Term Placenta

In a larger study to investigate gene expression in human placentae, we performed double-stranded RNA-Seq on 96 (35 early and 61 term gestation) samples with an average of ~35.8 million paired-end reads per sample. For the purposes of the current study, the 35 early gestation samples were excluded, and 1 term sample was removed due to ambiguous labelling, leaving a total of 50 [26 samples from the SCOPE cohort (11 male, 15 female) and 24 samples from the STOP cohort (9 male, 15 female)] samples for downstream analyses. FASTQ files were aligned to human genome GRCh37 (hs37d5) using STAR (35) and RNA-Seq data was summarised to the gene level using featureCounts (36) from which we detected 20654 genes with an official gene symbol. After removing genes from the X- and Y-Chromosomes, and mitochondrial genes (706 genes removed), and filtering for genes with low expression (<2 counts per million in 6 samples), 12903 genes remained for downstream analyses.

**Figure 1:**
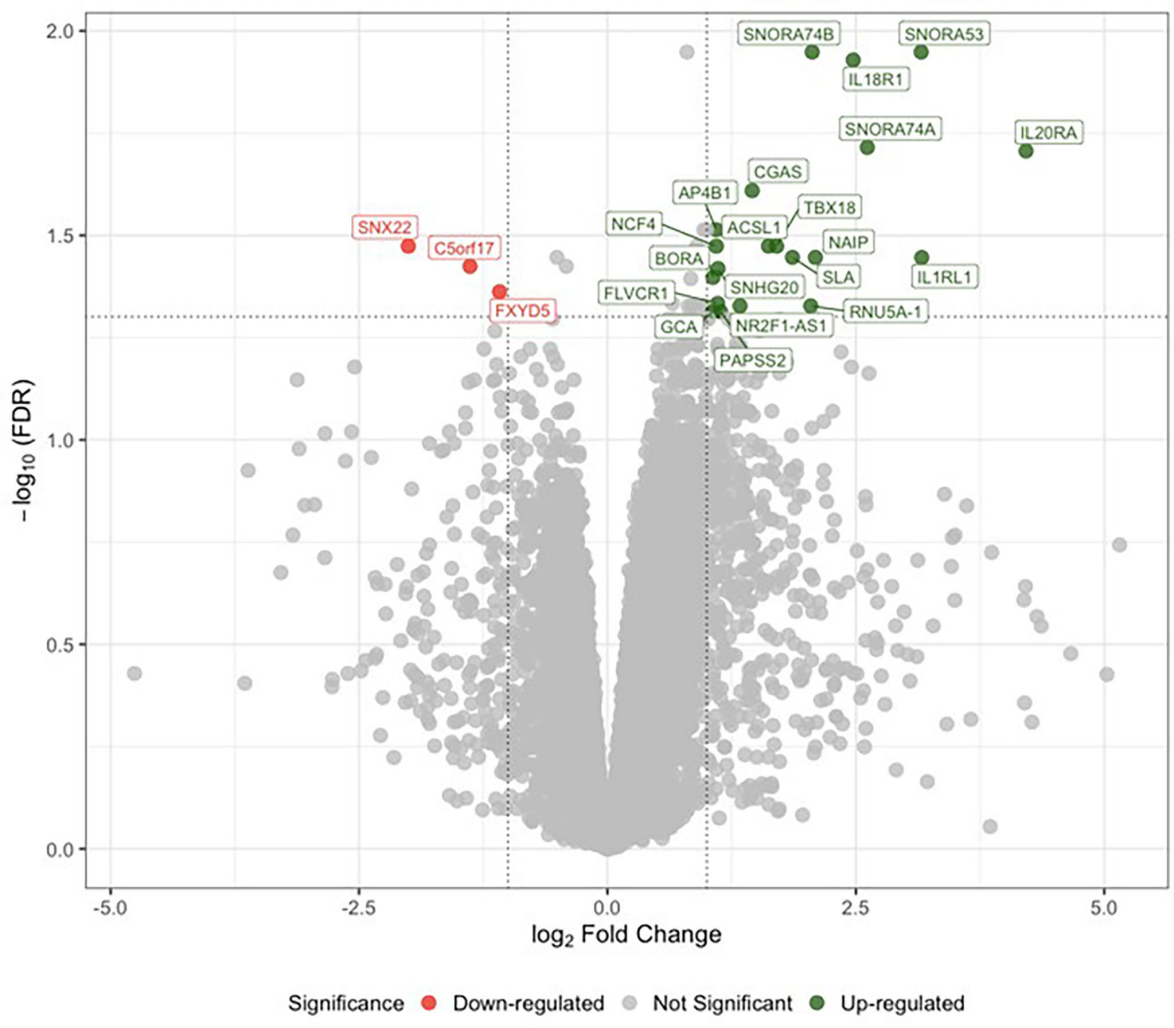
Analysis of differential gene expression determined by RNA sequencing between labouring lower segment Caesarean section (LLSCS) (n = 7) and prelabour lower segment Caesarean section (PLSCS) (n = 3) in male-bearing pregnancies only. Volcano plot shows the level of change (log transformed normalised counts); differentially expressed genes (false discovery rate (FDR) < 0.05 & log fold change (logFC) > |1|) indicated in red and green. Horizontal dotted line corresponds to FDR of 0.05 (-log_10_ scaled). Vertical dotted lines correspond to logFC > |1|.

Sex-specific differential expression analysis between male LLSCS and PLSCS showed 33 up- and 5 down-regulated genes (lower expression in LLSCS: FDR < 0.05) including up-regulation of *IL18R1*, *IL1RL1*, *IL20RA* (**Figure 1**). There were no significantly differentially expressed genes in the female comparison. It is important to note that the RNA-Seq differential expression experiment was performed to guide targets of our PCR experiments. The small number of samples used in this comparison, male LLSCS (n = 7), male PLSCS (n = 3) limit the stand-alone interpretation of these results.

### Markers of Inflammasome Activation, IL-1β and IL-18, Are Significantly Increased at mRNA Level in LLSCS Births

**Figure 2:**
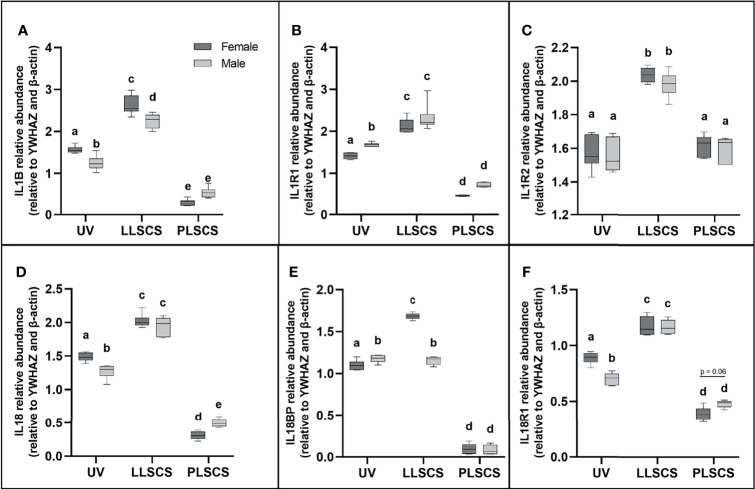
The mRNA expression of **(A)** Interleukin 1β (*IL1B*), **(B)** Interleukin 1 Type 1 Receptor (*IL1R1*), **(C)** Interleukin 1 Type 2 Receptor (*IL1R2*), **(D)** Interleukin 18 (*IL18*), **(E)** Interleukin 18 Binding Protein (*IL18BP*) and **(F)** Interleukin 18 Type 1 Receptor (*IL18R1*) in placentae from female- and male-bearing pregnancies classed by delivery mode (as determined by qPCR). Data are presented as a 10-90 percentile interleaved box-and-whisker plot. The same letter above bars indicates that groups are not different from each other. A different letter above bars indicates that groups are different (all *p* < 0.05). Dark grey colour denotes placenta samples from female fetal sex; light grey colour denotes placenta samples from male fetal sex. (Number of samples: unassisted vaginal (UV) 20/fetal sex; labouring lower segment Caesarean section (LLSCS) = 10/fetal sex; prelabour lower segment Caesarean section (PLSCS) = 5/fetal sex).

Activation of any inflammasome complex results in the production of mature IL-1β and IL-18 pro-inflammatory cytokines. Expression of *IL1B* and *IL18* mRNA (**Figures 2A** **, D**, respectively) was significantly upregulated in placentae from LLSCS births compared with UV for both female- (*p_IL1B_ <* 0.0001; *p_IL18_ <* 0.0001) and male-bearing (*p_IL1B_ <* 0.0001; *p_IL18_ <* 0.0001) pregnancies, as well as in placentae from LLSCS births compared with PLSCS for both female- (*p_IL1B_ <* 0.0001; *p_IL18_ <* 0.0001) and male-bearing (*p_IL1B_ <* 0.0001; *p_IL18_ <* 0.0001) pregnancies. Expression of *IL1B* and *IL18* mRNA was also upregulated in UV births compared with PLSCS for both female- (*p_IL1B_ <* 0.0001; *p_IL18_ <* 0.0001) and male-bearing (*p_IL1B_ <* 0.0001; *p_IL18_ <* 0.0001) pregnancies.

Expression of both *IL1B* and *IL18* mRNA was significantly decreased in placentae delivered *via* UV method in male compared to female-bearing pregnancies (*p_IL1B_ =* 0.0002; *p_IL18_ =* 0.0018). *IL1B* mRNA expression was significantly decreased in placentae delivered *via* LLSCS method in male- compared to female-bearing pregnancies (*p* < 0.0001) and *IL18* mRNA expression was significantly decreased in placentae delivered *via* PLSCS method in female- compared to male-bearing pregnancies (*p* = 0.0132).

### Receptors for IL-1β and IL-18 Are Significantly Increased in LLSCS Births

IL-1β can exert its inflammatory effects by binding to the IL1R1. *IL1R1* mRNA expression (**Figure 2B**) was significantly increased in placentae from LLSCS births compared with UV for both female- and male-bearing pregnancies, as well as from LLSCS births compared with PLSCS for both female- and male-bearing pregnancies and from UV births compared with PLSCS for both female- and male-bearing pregnancies (all *p* < 0.0001).

*IL1R1* mRNA expression was significantly increased in placentae delivered *via* UV birth in male- compared to female-bearing pregnancies (*p* = 0.0348).

IL-1β binding to the IL1R2 can have anti-inflammatory effects. *IL1R2* mRNA expression (**Figure 2C**) was significantly increased in placentae from LLSCS births compared with UV for both female- (*p <* 0.0001) and male-bearing (*p <* 0.0001) pregnancies, as well as from LLSCS births compared with PLSCS for both female- (*p <* 0.0001) and male-bearing (*p <* 0.0001) pregnancies.

IL18BP is a potent inhibitor of IL-18 activity. *IL18BP* mRNA expression (**Figure 2E**) was significantly increased in placentae from LLSCS births compared with UV for female-bearing (*p <* 0.0001) pregnancies only, as well as from LLSCS births compared with PLSCS for both female- (*p <* 0.0001) and male-bearing (*p <* 0.0001) pregnancies. *IL18BP* mRNA expression was significantly increased in placentae from UV births compared with PLSCS for both female- (*p <* 0.0001) and male-bearing (*p <* 0.0001) pregnancies.

*IL18BP* mRNA expression was significantly increased in placentae delivered *via* LLSCS method in female- compared to male-bearing pregnancies (*p* < 0.0001) and significantly decreased in placentae delivered *via* UV method in female- compared to male-bearing pregnancies (*p* = 0.0408).

Finally, IL18R1 is the receptor by which IL-18 binding exerts inflammatory effects. *IL18R1* mRNA expression (**Figure 2F**) was significantly increased in placentae from LLSCS births compared with UV for both female- (*p <* 0.0001) and male-bearing (*p <* 0.0001) pregnancies, as well as from LLSCS births compared with PLSCS for both female- (*p <* 0.0001) and male-bearing (*p <* 0.0001) pregnancies. *IL18R1* mRNA expression was significantly increased in placentae from UV births compared with PLSCS for both female- (*p <* 0.0001) and male-bearing (*p <* 0.0001) pregnancies.

*IL18R1* mRNA expression was significantly increased in placentae delivered *via* UV in female- compared to male-bearing pregnancies (*p* < 0.0001).

### Expression of Pro-Inflammatory *IL6, IL2RB, IL17RE, TNFAIP2, TNFSF10*, and *TNFRSF8* Is Increased in LLSCS Births

**Figure 3:**
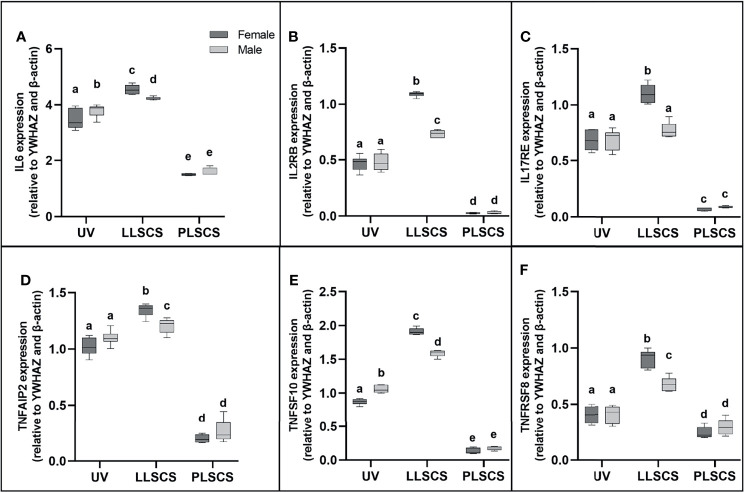
**(A)** Interleukin 6 (*IL6*), **(B)** Interleukin 2 Receptor β (*IL2RB*), **(C)** Interleukin 17 Receptor E (*IL17RE*), **(D)** Tumour Necrosis Factor Alpha Induced Protein 2 (*TNFAIP2*), **(E)** Tumour Necrosis Factor Super Family Member 10 (*TNFSF10*) and **(F)** Tumour Necrosis Factor Receptor Super Family Member 8 (*TNFRSF8*) mRNA expression in placentae from female- and male-bearing pregnancies classed by delivery mode (qPCR). Data are presented as a 10-90 percentile interleaved box-and-whisker plot. The same letter above bars indicates that groups are not different from each other. A different letter above bars indicates that groups are different (all *p* < 0.05). Dark grey colour denotes placenta samples from female fetal sex; light grey colour denotes placenta samples from male fetal sex. (Number of samples: unassisted vaginal (UV) 20/fetal sex; labouring lower segment Caesarean section (LLSCS) = 10/fetal sex; prelabour lower segment Caesarean section (PLSCS) = 5/fetal sex).

IL-6 is commonly used as a marker of inflammation. *IL6* mRNA expression (**Figure 3A**) was significantly increased in placentae from LLSCS births compared with UV for female- (*p <* 0.0001) and male-bearing (*p* = 0.0035) pregnancies, as well as from LLSCS births compared with PLSCS for both female- (*p <* 0.0001) and male-bearing (*p <* 0.0001) pregnancies, and from UV births compared with PLSCS for both female- (*p <* 0.0001) and male-bearing (*p <* 0.0001) pregnancies.

*IL6* mRNA expression was significantly increased in placentae delivered *via* LLSCS in female- compared to male-bearing pregnancies (*p* = 0.0392) and significantly decreased in placentae delivered vaginally in female- compared to male-bearing pregnancies (*p* = 0.0359).

IL2RB (IL-2Rβ) refers to the β subunit of the interleukin-2 receptor. It is involved in the inflammatory response by activating T and NK cell subsets (37). Specifically, the human IL2RB gene has been shown to use an LTR alternative promoter to drive expression, specifically in the placenta (38). *IL2RB* mRNA expression (**Figure 3B**) was significantly increased in placentae from LLSCS births compared with UV for female- (*p <* 0.0001) and male-bearing (*p* < 0.0001) pregnancies, as well as from LLSCS births compared with PLSCS for both female- (*p <* 0.0001) and male-bearing (*p <* 0.0001) pregnancies and from UV births compared with PLSCS for both female- (*p <* 0.0001) and male-bearing (*p <* 0.0001) pregnancies.

*IL2RB* mRNA expression was significantly increased in placentae delivered *via* LLSCS method in female- compared to male-bearing pregnancies (*p* < 0.0001).

IL17RE is part of the IL-17C pathway and functions as a pivotal regulator of innate immunity (39). Interestingly, IL-17C induces inflammation, but also promotes tissue healing (40). *IL17RE* mRNA expression (**Figure 3C**) was significantly increased in placentae from LLSCS births compared with UV for female-bearing (*p <* 0.0001) pregnancies only, as well as from LLSCS births compared with PLSCS for both female- (*p <* 0.0001) and male-bearing (*p <* 0.0001) pregnancies; and from UV births compared with PLSCS for both female- (*p <* 0.0001) and male-bearing (*p <* 0.0001) pregnancies.

*IL17RE* mRNA expression was significantly increased in placentae delivered *via* LLSCS in female- compared to male-bearing pregnancies (*p* < 0.0001).

TNFAIP2 expression is induced by other cytokines, including IL-1β, and plays essential roles in inflammation as a primary response gene (41), as well as angiogenesis and cell migration (42). *TNFAIP2* mRNA expression (**Figure 3D**) was significantly increased in placentae from LLSCS births compared with UV for female- (*p <* 0.0001) and male-bearing (*p* = 0.0443) pregnancies, as well as from LLSCS births compared with PLSCS for both female- (*p <* 0.0001) and male-bearing (*p <* 0.0001) pregnancies and from UV births compared with PLSCS for both female- (*p <* 0.0001) and male-bearing (*p <* 0.0001) pregnancies.

*TNFAIP2* mRNA expression was significantly increased in placentae delivered *via* LLSCS method in female- compared to male-bearing pregnancies (*p* = 0.0071).

TNFSF10 is a cytokine belonging to the TNF-ligand family. The main role for TNFSF10 is in apoptosis of mutated cells, as it does not kill non-malignant cells (43). However, it is also expressed as an inflammatory marker in most healthy tissues. *TNFSF10* mRNA expression (**Figure 3E**) was significantly increased in placentae from LLSCS births compared with UV for female- (*p <* 0.0001) and male-bearing (*p* = 0.0035) pregnancies, as well as from LLSCS births compared with PLSCS for both female- (*p <* 0.0001) and male-bearing (*p <* 0.0001) pregnancies and from UV births compared with PLSCS for both female- (*p <* 0.0001) and male-bearing (*p <* 0.0001) pregnancies.

*TNFSF10* mRNA expression was significantly increased in placentae delivered *via* LLSCS in female- compared to male-bearing pregnancies (*p* < 0.0001) and significantly decreased in placentae delivered *via* UV method in female- compared to male-bearing pregnancies (*p* < 0.0001).

Finally, TNFRSF8 (another member of the TNF-receptor superfamily) is a marker of inflammation as it is thought to be expressed only on activated T and B cells, not on resting cells. Activation of this receptor leads to signaling *via* the NF-κB pathway. *TNFRSF8* mRNA expression (**Figure 3F**) was significantly increased in placentae from LLSCS births compared with UV for female- (*p <* 0.0001) and male-bearing (*p* < 0.0001) pregnancies, as well as from LLSCS births compared with PLSCS for both female- (*p <* 0.0001) and male-bearing (*p <* 0.0001) pregnancies; and from UV births compared with PLSCS for both female- (*p =* 0.0020) and male-bearing (*p =* 0.0316) pregnancies.

*TNFRSF8* mRNA expression was significantly increased in placentae delivered *via* LLSCS in female- compared to male-bearing pregnancies (*p* < 0.0001).

### Expression of Transcription Factors STAT1 and STAT2 Is Increased in Female UV Births Only

**Figure 4:**
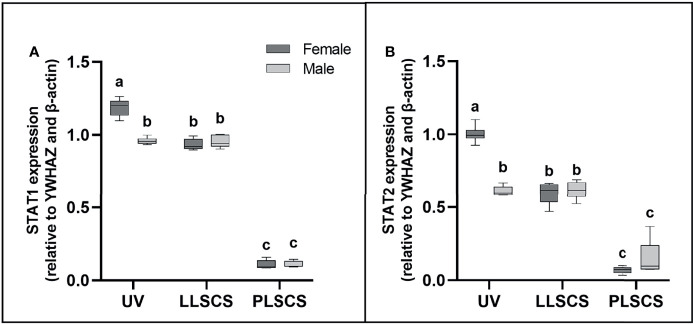
**(A)** Signal transducer and activator of transcription 1 (*STAT1*) and **(B)** 2 (*STAT2*) mRNA expression (qPCR) in placentae from female- and male-bearing pregnancies classed by delivery mode. Data are presented as a 10-90 percentile interleaved box-and-whisker plot. The same letter above bars indicates that groups are not different from each other. A different letter above bars indicates that groups are different (all *p* < 0.05). Dark grey colour denotes placenta samples from female fetal sex; light grey colour denotes placenta samples from male fetal sex. (Number of samples: unassisted vaginal (UV) 20/fetal sex; labouring lower segment Caesarean section (LLSCS) = 10/fetal sex; prelabour lower segment Caesarean section (PLSCS) = 5/fetal sex).

STAT1 and STAT2 are transcription factors which mediate type I and III interferon signalling *via* the JAK-STAT pathway; as such they play essential roles in the adaptive immune response (44). *STAT1* and *STAT2* mRNA expression (**Figures 4A** **, B**, respectively) was significantly increased in placentae from UV births compared with LLSCS for female-bearing (*p_STAT1_ <* 0.0001; *p_STAT2_ <* 0.0001) pregnancies only, as well as from LLSCS births compared with PLSCS for both female- (*p <* 0.0001) and male-bearing (*p <* 0.0001) pregnancies and from UV births compared with PLSCS for both female- (*p <* 0.0001) and male-bearing (*p <* 0.0001) pregnancies.

*STAT1* and *STAT2* mRNA expression was significantly increased in placentae delivered *via* UV delivery in female- compared to male-bearing pregnancies (*p_STAT1_ <* 0.0001; *p_STAT2_ <* 0.0001).

### Expression of Anti-Inflammatory Interleukin IL-10 Is Increased in Male LLSCS Births Only

**Figure 5:**
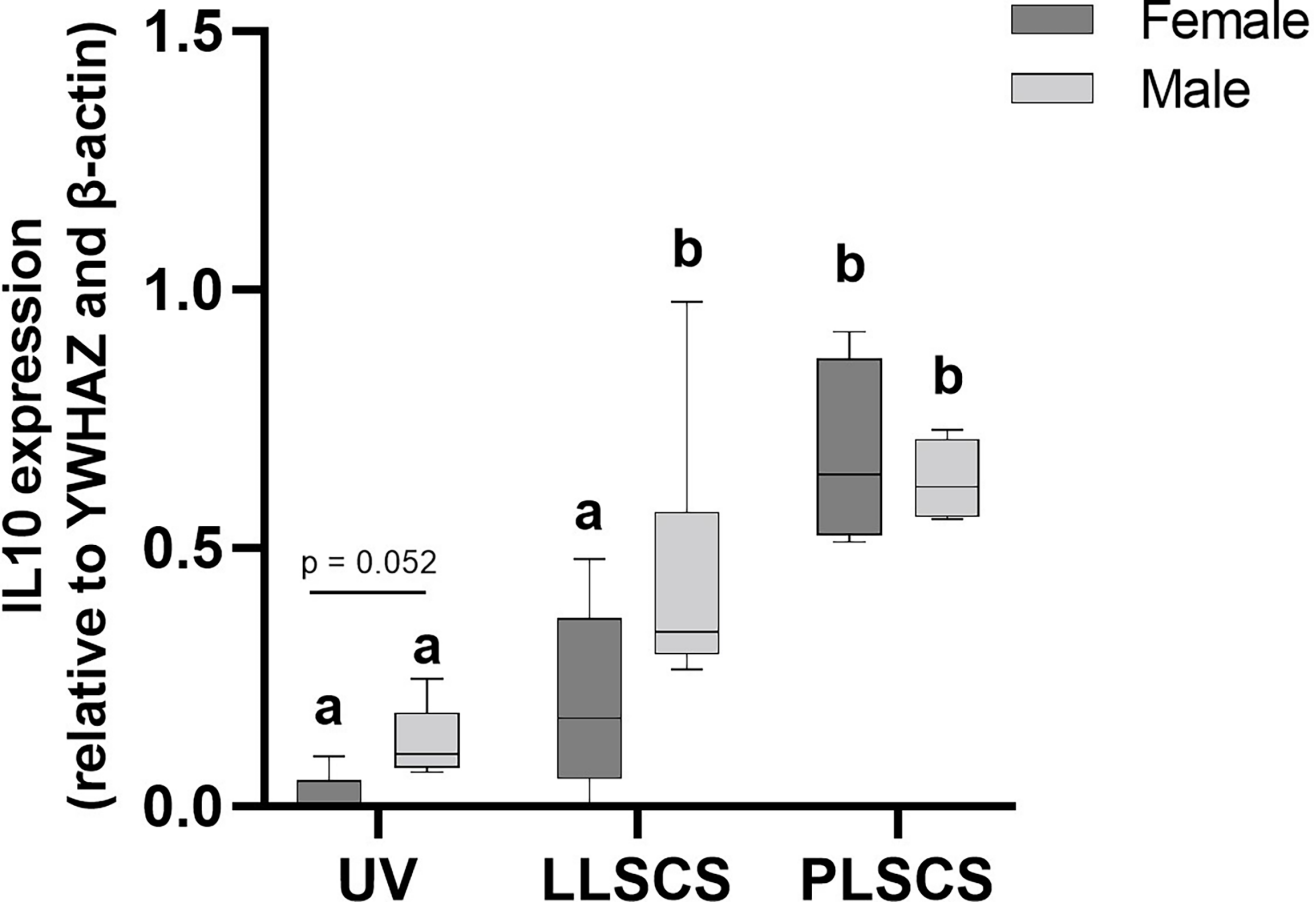
The mRNA expression (as determined by qPCR) of Interleukin 10 (*IL10*) in placentae from female- and male-bearing pregnancies classed by delivery mode. Data are presented as a 10-90 percentile interleaved box-and-whisker plot. The same letter above bars indicates that groups are not different from each other. A different letter above bars indicates that groups are different (all *p* < 0.05). Dark grey colour denotes placenta samples from female fetal sex; light grey colour denotes placenta samples from male fetal sex. (Number of samples: unassisted vaginal (UV) 20/fetal sex; labouring lower segment Caesarean section (LLSCS) = 10/fetal sex; prelabour lower segment Caesarean section (PLSCS) = 5/fetal sex).

IL-10 is a cytokine with potent anti-inflammatory roles; indeed, absence of IL-10 leads to immunopathologies that are detrimental to the host, without any effect on pathogen load (45–47). *IL10* mRNA expression (**Figure 5**) was significantly increased in placentae from LLSCS births compared with UV for male-bearing (*p =* 0.0063) pregnancies only, as well as from LLSCS births compared with PLSCS for female-bearing (*p =* 0.0003) pregnancies only, and from UV births compared with PLSCS for both female- (*p <* 0.0001) and male-bearing (*p =* 0.0001) pregnancies.

*IL10* mRNA expression was significantly increased in placentae delivered *via* LLSCS method in male- compared to female-bearing pregnancies (*p =* 0.0474).

### Expression of Alarmin IL-33, and Receptor IL1RL1, Is Increased in Male LLSCS Births Only

**Figure 6:**
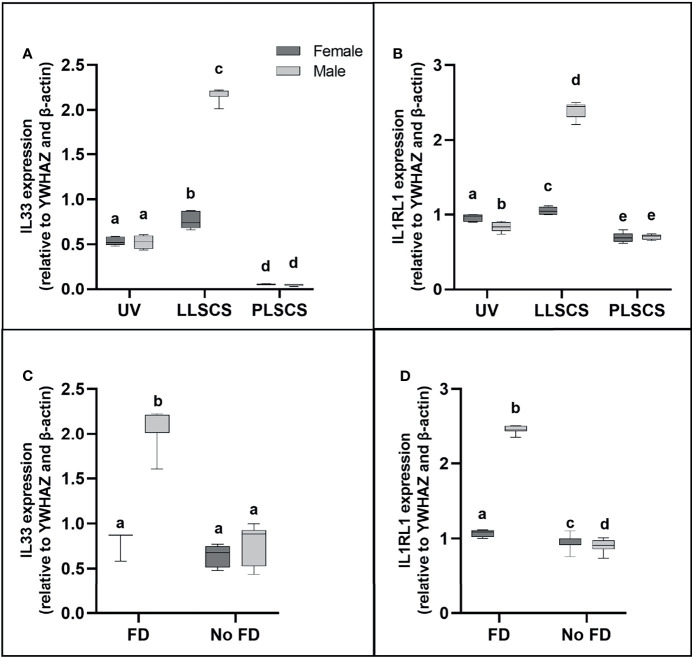
**(A)** Interleukin 33 (*IL33*) and **(B)** Interleukin 1 Receptor Like 1 (*IL1RL1*) mRNA expression in placentae from female- and male-bearing pregnancies classed by delivery mode, and by indication of fetal distress (FD) [**(C)** IL33 in FD, **(D)** IL1RL1 in FD)] during labour (qPCR). Data are presented as a 10-90 percentile interleaved box-and-whisker plot. The same letter above bars indicates that groups are not different from each other. A different letter above bars indicates that groups are different (all *p* < 0.05). Dark grey colour denotes placenta samples from female fetal sex; light grey colour denotes placenta samples from male fetal sex. (Number of samples: unassisted vaginal (UV) 20/fetal sex; labouring lower segment Caesarean section (LLSCS) = 10/fetal sex; prelabour lower segment Caesarean section (PLSCS) = 5/fetal sex. Samples with FD = 9, samples with no FD = 51).

IL-33 (a member of the IL1 family) is mainly secreted by damaged barrier cells as an alarmin cytokine (48); that is, it is used as an alarm to signal cellular damage or stress. Its receptor, IL1RL1, can be membrane-bound or soluble, and acts as an inhibitor of IL-33 functioning as a decoy receptor (49). *IL33* and *IL1RL1* mRNA expression (**Figures 6A** **, B**, respectively) was significantly increased in placentae from LLSCS births compared with UV for female- (*p_IL33_* < 0.0001; *p_IL1RL1_* = 0.0112) and male-bearing (*p_IL33_* < 0.0001; *p_IL1RL1_* < 0.0001) pregnancies, as well as from LLSCS births compared with PLSCS for female- (*p_IL33_* < 0.0001; *p_IL1RL1_* < 0.0001) and male-bearing (*p_IL33_* < 0.0001; *p_IL1RL1_* < 0.0001) pregnancies, and from UV births compared with PLSCS for both female- (*p_IL33_* < 0.0001; *p_IL1RL1_* < 0.0001) and male-bearing (*p_IL33_* < 0.0001; *p_IL1RL1_* = 0.0004) pregnancies.

*IL33* and *IL1RL1* mRNA expression was significantly increased in placentae delivered *via* LLSCS in male- compared to female-bearing pregnancies (*p_IL33_* < 0.0001; *p_IL1RL1_* < 0.0001). *IL1RL1* mRNA expression was also significantly decreased in placentae delivered *via* UV delivery in male- compared to female-bearing pregnancies (*p* = 0.0007).

### Expression of Alarmin *IL33*, and Receptor *IL1RL1* Is Only Increased in Male LLSCS Births With Fetal Distress During Labour

Fetal distress (FD) was ascertained from clinical notes indicating meconium presence in the amniotic fluid, and/or obstetrician’s decision to use a Ventouse cap or forceps, or proceed to LLSCS due to failure to progress. Interestingly, *IL33* expression (**Figure 6C**) was only significantly increased in placentae from male babies with FD compared with births where no FD was detected (*p* < 0.0001), regardless of delivery mode. *IL1RL1* mRNA expression (**Figure 6D**) was significantly increased in placentae from both female- (*p* = 0.0329) and male-bearing (*p* < 0.0001) births with FD compared with births where no FD was detected.

*IL33* and *IL1RL1* mRNA expression was significantly increased in placentae from male- compared to female-bearing deliveries where FD was detected (*p_IL33_* < 0.0001; *p_IL1RL1_* < 0.0001). *IL1RL1* mRNA expression was also significantly decreased in placentae from male- compared to female-bearing deliveries where no FD was detected (*p* = 0.0129).

### Expression of IL-1β Protein Is Significantly Increased in LLSCS Females Only

**Figure 7:**
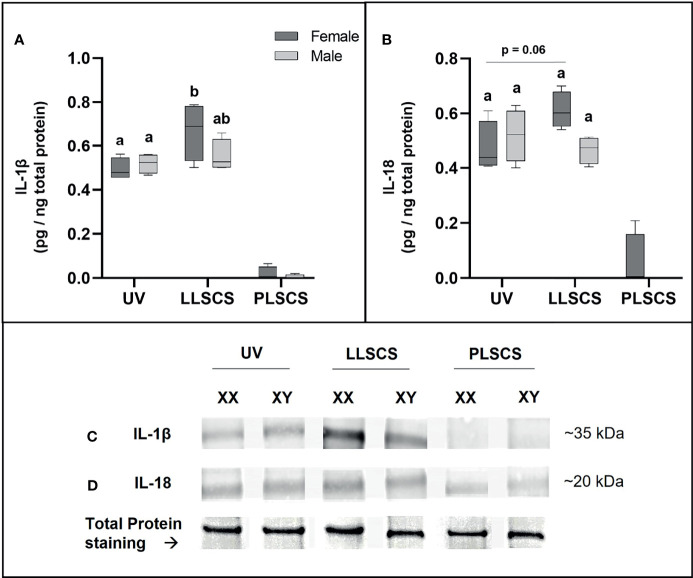
The mRNA expression of Interleukin 1β (IL-1β) **(A, C)** and Interleukin 18 (IL-18) **(B, D)** protein in placentae from female- and male-bearing pregnancies classed by delivery mode. For **(A, B)**, Data are presented as a 10-90 percentile interleaved box-and-whisker plot. The same letter above bars indicates that groups are not different from each other. A different letter above bars indicates that groups are different (all *p* < 0.05). Dark grey colour denotes placenta samples from female fetal sex; light grey colour denotes placenta samples from male fetal sex. **(C, D)** show representative images of staining with an IL-1β antibody (molecular weight ~35 kDa) and an IL-18 antibody (molecular weight ~20 kDa), respectively. Below this is a representative image of stain-free Total Protein staining as a control. (Number of samples: unassisted vaginal (UV) = 6/fetal sex; labouring lower segment Caesarean section (LLSCS) = 4/fetal sex; prelabour lower segment Caesarean section (PLSCS) = 2/fetal sex).

IL-1β protein expression (**Figures 7A** **, C**) was significantly increased in placentae from LLSCS births compared with UV for female-bearing pregnancies only (*p* = 0.0031).

IL-18 protein expression (**Figures 7B** **, D**) was higher in placentae from LLSCS births compared with UV for female-bearing pregnancies only but was not statistically significant (*p* = 0.0602).

IL-1β and IL-18 protein expression in placentae from PLSCS births is illustrated in **Figure 7**, however statistical analyses could not be performed due to low sample numbers (n = 2/fetal sex).

## Discussion

This study has shown that there is a general increase in inflammasome activation (as evidenced by the expression of genes encoding IL-1β and IL-18), and inflammatory molecule expression, in placentae from LLSCS births compared to UV and PLSCS births. This result was consistent with our hypothesis that, as emergency caesarean sections in labour are conducted due to maternal or fetal distress, placental gene expression in LLSCS births would reflect increased inflammation.

Inflammasome activation, and inflammatory molecule expression, is also increased in placentae from LLSCS compared with PLSCS births, and in UV compared with PLSCS deliveries. This was also consistent with our hypothesis that placentae from PLSCS births would show very low levels of inflammation compared with other modes of delivery, given that they do not experience the inflammatory response associated with labour. However, whilst we did not see the degree of inflammatory molecule expression that was observed in labouring births, there was some inflammatory molecule expression in placentae from PLSCS births as would be expected. Although all of the women with PLSCS in our study had uncomplicated pregnancies, these deliveries are often scheduled due to a pregnancy complication such as preeclampsia or gestational diabetes.

The initiation of labour is reliant on the functional withdrawal of progesterone and a significant increase in myometrial progesterone receptor A (PR-A), leading to an overall rise in the PR-A/PR-B expression ratio (50). This functional withdrawal allows estrogen signalling to dominate inducing upregulation of contraction-associated proteins (CAPs), prostaglandins, oxytocin receptors in the myometrium, and collagenases and metalloproteinases in the cervix that cause its ripening. These uterotonins lead to coordinated uterine contractions. Whilst the exact mechanism inducing progesterone withdrawal is not completely understood, progesterone has long been known to be anti-inflammatory and maintains myometrial quiescence. Upon progesterone withdrawal, prostaglandins have also been implicated in labour (51), potentially by establishing a positive-feedback loop in the uterus with prostaglandins and inflammatory cytokines released by infiltrating leukocytes in the myometrium (3, 52). Furthermore, an association has long been established between inflammatory cell infiltration of the fetal membranes and decidua in labour and release of increased prostaglandins and leukotrienes (53, 54). Indeed, the fetal membranes are essential for allowing glucocorticoid signalling, leading to stimulation of prostaglandin production (55).

Whilst parturition is associated with an influx of leukocytes into the myometrium (26), this same phenomenon has been observed in the decidua (56) as well as in the cervix, where they potentially play a role in cervical ripening (57). Previous studies have shown that these leukocytes express pro-inflammatory cytokines IL-1β, IL-18 and IL-6 (58), and while inflammatory cells have not been detected in the placenta during parturition, this increase in pro-inflammatory cytokines that is observed in the myometrium and uterus is also in the chorio-decidua (26), as well as in amniotic fluid (59). Our data imply that expression of inflammatory cytokines and inflammation observed in the placenta following delivery could serve as an indication of molecular changes occurring within the uterus.

Not only did this study find that pro-inflammatory cytokines are increased in the placenta in labour compared to no labour, but patterns of expression were also noted which were fetal sex specific (**Table 3**). The expression of genes encoding inflammatory cytokines IL-1β and IL-18 was higher in placentae from female bearing pregnancies compared to male, indicating increased inflammasome activation in females.

**Table 3:** Expression patterns of inflammasome and inflammatory molecules in placentae from female and male-bearing pregnancies, classified by mode of delivery.

	UV(female vs male)	LLSCS(female vs male)	PLSCS(female vs male)	LLSCS vs UV	LLSCS vs PLSCS	PLSCS vs UV
IL1β	↑	↑	–	↑ both	↑ both	↓ both
IL1R1	↑	–	–	↑ both	↑ both	↓ both
IL1R2	–	–	–	↑ both	↑ both	–
IL18	↑	–	↑	↑ both	↑ both	↓ both
IL18BP	↑	↑	–	↑ only	↑ both	↓ both
IL18R1	↑	–	–	↑ both	↑ both	↓ both
IL6	↑	↑	–	↑ both	↑ both	↓ both
IL2RB	–	↑	–	↑ both	↑ both	↓ both
IL17RE	–	↑	–	↑ only	↑ both	↓ both
TNFAIP2	–	↑	–	↑ both	↑ both	↓ both
TNFSF10	–	↑	–	↑ both	↑ both	↓ both
TNFRSF8	–	↑	–	↑ both	↑ both	↓ both
STAT1	↑	–	–	↓ only	↑ both	↓ both
STAT2	↑	–	–	↓ only	↑ both	↓ both
IL10	–	↑	–	↑ only	↑ only	↑ both
IL33	–	↑	–	↑ both	↑ both	↓ both
IL1RL1	↑	↑	–	↑ both	↑ both	↓ both

**Orange** indicates a result seen only in placentae from female-bearing pregnancies. **Blue** indicates a result seen only in placentae from male-bearing pregnancies. Both indicates altered expression in both female and male placentae. UV, unassisted vaginal; LLSCS, labouring lower sagittal Caesarean section; PLSCS, prelabour lower sagittal Caesarean section.

**Figure 8:**
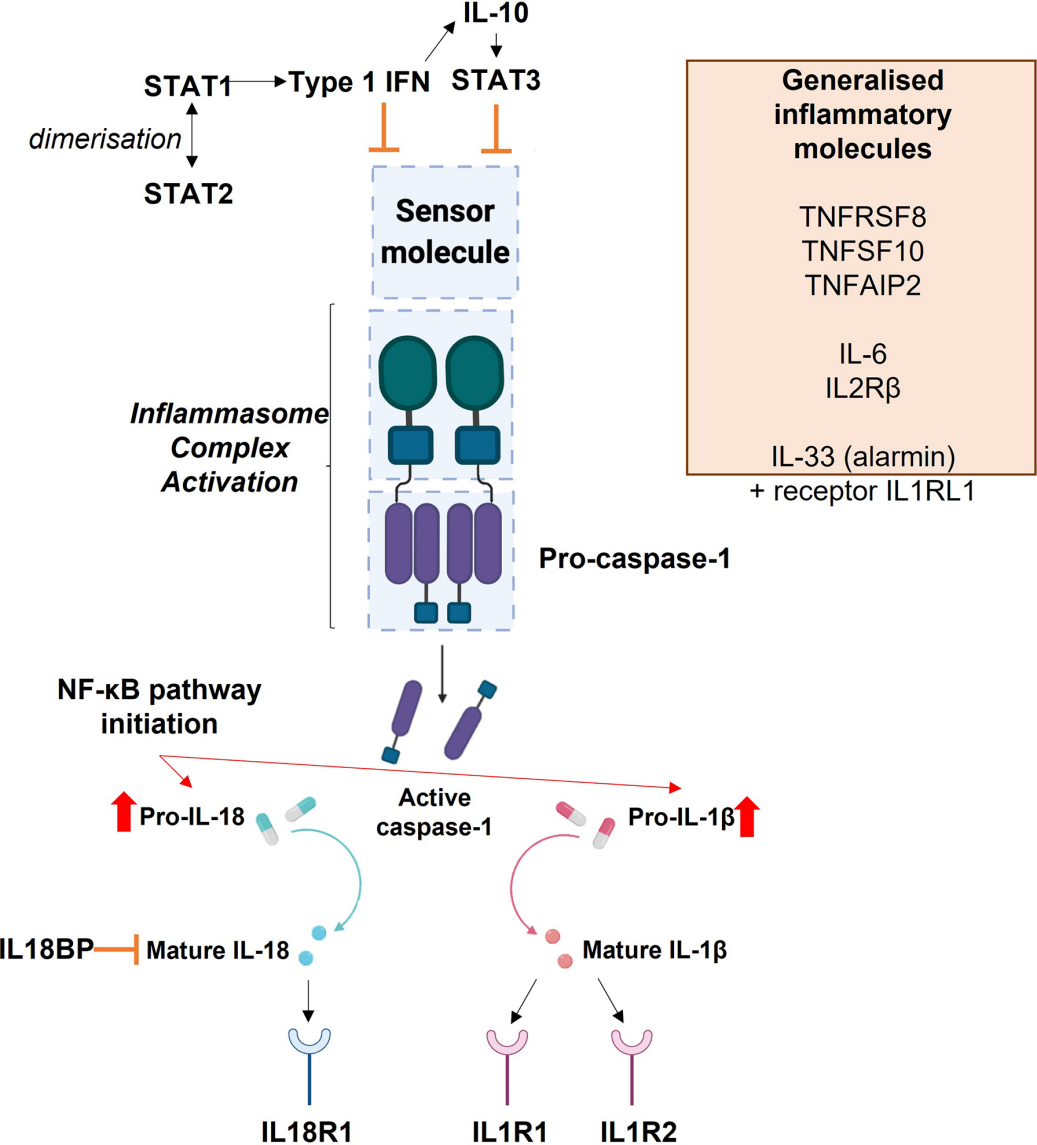
Diagrammatic representation of the interactions of inflammasome and inflammatory molecules explored in this study.

Many inflammatory genes and/or receptors (*IL1B, IL6, IL2RB, IL17RE, TNFAIP2, TNFSF10* and *TNFRSF8*; for interactions see **Figure 8**) also followed a pattern of upregulation in placentae from LLSCS compared with UV and PLSCS births, but also were significantly upregulated in placentae from female compared to male bearing LLSCS births. These clearly indicate heightened inflammation in placentae from female-bearing LLSCS births. Interestingly, *IL18BP* mRNA expression was also significantly increased in placentae from female-bearing LLSCS births compared with UV births, where this was not observed in males. As IL18BP functions as an inhibitor of IL-18 activity by competitively binding to IL-18, impeding IL-18/IL18R1 binding, it is classified as an anti-inflammatory molecule. The upregulation of IL18BP in female but not male-bearing placentae could indicate a strategy to minimize damage from a large inflammatory response, given the clearly larger inflammatory reaction observed in placentae from female compared to male-bearing LLSCS births. This is important as excess inflammasome activation is associated with extensive pyroptosis (60). However, in the placenta it is not known what impact this may have for these female babies.

In addition to the relatively lower expression of inflammasome and inflammatory molecules in placentae from male compared with female-bearing LLSCS births, anti-inflammatory cytokine *IL10* mRNA expression was significantly increased. IL-10 plays an essential role in preventing pathological inflammation (45), as well as preventing chronic inflammatory conditions (61). In fact, the levels of *IL10* expression in placentae from male-bearing LLSCS births was no different to that in PLSCS births, potentially controlling the inflammatory response in LLSCS births to ensure there is not overactivation of the inflammasome. This was not observed in female-bearing placentae, which had the same level of *IL10* expression in LLSCS and UV births.

Furthermore, *IL33* and *IL1RL1* mRNA were significantly upregulated by approximately 2.5-fold in placentae from male compared with female-bearing LLSCS births. As briefly mentioned above, IL-33 does not function as a typical inflammatory cytokine but instead as an alarmin, providing an indication to surrounding tissue of damage or stress.

Previous studies have identified that male-bearing pregnancies are more likely to end in LLSCS compared with female-bearing (62); however, this is not due to failure to progress through labour but instead due to signs of fetal distress (63). In conjunction with our data, this could indicate that placentae from male-bearing LLSCS births upregulate IL-33 as a signal of fetal distress, inducing IL1RL1 expression and mediating inflammation. This is not seen in placentae from female-bearing LLSCS births, where the magnitude of the *IL33* expression is significantly reduced, and inflammatory markers are more highly expressed compared with males. Indeed, when samples were separated by clinical notes indicating fetal distress, a large increase in *IL33* and *IL1RL1* expression was observed in placentae from male, but not female-bearing births, suggesting enhanced signalling for damage and stress in problematic male-bearing labours compared with female-bearing.

A major limitation of this study is its opportunistic nature that relied on data from a larger unrelated study that profiled the placental transcriptome across gestation. Data for uncomplicated pregnancies at term for whom mode of delivery was known were selected. Therefore, the data may not represent the whole population. Furthermore, data was available for a relatively small number of placentae from PLSCS births. Information about intra-partum medications were not available so data may be influenced by these for both emergency and planned caesarean delivery. Moreover, all data are from analysis of chorionic villus samples, and the specific cell types present in these samples were not quantified. Future studies should be larger and designed specifically to determine differences in the inflammasome between different modes of delivery with attention to all details of the labour and delivery including medications, analgesia and anaesthesia.

Overall, increased activation of the placental inflammasome in LLSCS, which is often a life-saving intervention, likely also indicates greater exposure of the fetus during labour and delivery to inflammation. Perinatal inflammation and caesarean delivery are known to impact post-natal health of the infant. The former associates with brain injury in preterm labour and subsequent neurological disorders the best known of which is cerebral palsy (2).

Caesarean section not only has implications for maternal mortality, morbidity, recovery and future pregnancy but it also has been implicated in long term health and wellbeing for children [reviewed in (64)]. Specifically, children who were delivered by caesarean section are at greater risk of immune disorders such as asthma, atopy, allergy with evidence for an altered gut microbiome and potentially they are at risk of childhood obesity and metabolic disorders (64). A recent systematic review and meta-analysis of over 20 million births has demonstrated increased risk for autism spectrum disorders (ASD) and attention deficit hyperactivity disorder (ADHD) in children delivered by caesarean section with similar effects for both in labour and planned (65). Compression of the fetus as it traverses the birth canal causes a surge in glucocorticoids that is believed to be beneficial to the quick transition to extrauterine life that the neonate must make. Previously it was thought that negative effects of caesarean delivery are due to the lack of the big squeeze experienced during vaginal delivery. However, more recent thinking on the mechanisms of adverse effects of caesarean delivery on child development implicate altered epigenetic state and changes to the gut microbiome (66). Inflammation could be a factor in these. Here we have identified changes in placental expression of inflammatory mediators in both LLSCS and PLSCS deliveries. With further studies and follow up of children, these could potentially be used as indicators of perinatal exposure to inflammation that may impact child development, even for term deliveries where future risks to the child are generally not suspected.

To summarise our findings, placentae from female-bearing LLSCS deliveries had the highest inflammasome activation, followed by male-bearing LLSCS deliveries and UV deliveries. Placentae from PLSCS deliveries had the lowest inflammasome activation, though some expression of inflammatory genes was detected. Compared to babies delivered UV, the most common method of delivery, those delivered by LLSCS may be more at risk of inflammatory insult. The placental inflammasome may be useful for non-invasively identifying those babies most at risk. Conversely, PLSCS placentae reflect low level inflammation that may also disadvantage the baby who may not be optimally primed for their transition to extrauterine life, but this requires further investigation.

Our study shows that inflammation is indeed evident in the placenta in term uncomplicated UV births, accompanying the loss of immune tolerance at parturition. Not only is this necessary to mediate the labour cascade but inflammasome expression in the placenta without significant infiltration by maternal leukocytes may aid in placental separation, blood clotting and closure of maternal spiral arteries which are further compressed by uterine contractions following delivery of the placenta. We have certainly demonstrated that placental inflammasome activation at delivery reflects the end of tolerance. However, clearly the end of tolerance is not the same for all pregnancies and mode of delivery and its impact on the placental inflammasome are clear indicators of that. Placental inflammation associated with UV delivery may help not only labour but also the rapid transition to extrauterine life that babies must make. Reductions in the placental inflammasome in PLSCS may be associated with the reduced ease with which these babies, so delivered, make that transition.

Signal Transducer and Activator of Transcription 1 and 2 (STAT1 and STAT2) dimerise in response to Type 1 Interferon (IFN) signalling. This signalling can itself inhibit inflammasome activation or can inhibit inflammasome activation *via* Interleukin-10 (IL-10), which then leads to the production of STAT3. The blue boxes in the centre of the image depict the basic inflammasome activation pathway: a sensor molecule is stimulated which leads to activation of the inflammasome complex by pro-caspase-1. This leads to the production of active caspase-1, which promotes the conversion of pro-IL-1β and pro-IL-18 into their mature forms. The release of mature IL-1β and IL-18 signal activation of the inflammasome. Their release also initiates the NF-κB pathway. Mature IL-18 acts upon the IL-18 Receptor 1 (IL18R1), where its binding stimulates a pro-inflammatory response. Interleukin 18 Binding Protein (IL18BP) acts as an inhibitor of IL-18. Mature IL-1β can bind to the Interleukin 1 Type 1 Receptor (IL1R1) to initiate a pro-inflammatory response, or to the Interleukin 1 Type 2 Receptor (IL1R2) to induce anti-inflammatory activity. The orange box lists other inflammatory molecules explored within this study which are not specifically associated with this inflammasome activation pathway: Tumour Necrosis Factor Receptor Superfamily Member 8 (TNFRSF8), Tumour Necrosis Factor Superfamily Member 10 (TNFSF10), Tumour Necrosis Factor Alpha Induced Protein 2), Interleukin 6 (IL-6), Interleukin 2 Receptor β (IL2RB), Interleukin 33 (IL-33) and Interleukin 1 Receptor Like 1 (IL1RL1).

## Data Availability Statement

The data presented in the study are deposited in the Short reads Archive (SRA) repository, accession number PRJNA816267.

## Ethics Statement

The studies involving human participants were reviewed and approved by The Queen Elizabeth Hospital and Lyell McEwin Hospital Human Research Ethics Committee TQEH/LMH HREC/1712/5/2008 (SCOPE Study) Women’s and Children’s Network Human Research Ethics Committee HREC/14/WCHN/90 (STOP Study). The patients/participants provided their written informed consent to participate in this study.

## Author Contributions

AA conceptualised this work, performed experimental work and formal analysis of data, original draft preparation, and review and editing. MS optimised the methodology for, and performed, bioinformatic analysis of data, prepared visualisations, and reviewed and edited the manuscript. MH contributed to experimental work and review and editing. JB contributed to methodology optimisation of bioinformatic analysis. DM optimised the methodology for, and performed, experimental work. FT contributed to experimental work. SL contributed to statistical methodologies used in the paper, as well as review and editing. GD provided clinical expertise and edited the manuscript. TJ-K reviewed and edited the manuscript. CR provided supervision, funding acquisition, interpretation of data, review and editing of the manuscript. All authors contributed to the article and approved the submitted version.

## Funding

RNA sequencing data used in this study was funded by NIH NICHD R01 HD089685-01 *Maternal molecular profiles reflect placental function and development across gestation* PI Roberts, a National Health and Medical Research Council Investigator Grant (GNT1174971) awarded to CTR and a Matthew Flinders Professorial Fellowship awarded to CTR and funded by Flinders University. JB is supported by the James & Diana Ramsay Foundation.

## Conflict of Interest

The authors declare that the research was conducted in the absence of any commercial or financial relationships that could be construed as a potential conflict of interest.

## Publisher’s Note

All claims expressed in this article are solely those of the authors and do not necessarily represent those of their affiliated organizations, or those of the publisher, the editors and the reviewers. Any product that may be evaluated in this article, or claim that may be made by its manufacturer, is not guaranteed or endorsed by the publisher.
